# Rhisodontrypy, or Hullihen’s Operation

**Published:** 1895-11

**Authors:** S. B. Brown


### THE DENTAL REGISTER.

Vol. XLIX.] NOVEMBER, 1895. [No. 11.

### Communications.

### Rhisodontrypy, or Hullihen's Operation.

BY DR. S. B. BROWN, D.D.S.

Read before the Tri-State Dental Meeting at Detroit, June 17th, 1895.

Lessons from history awaken and stimulate successive generations to a nobler endeavor.

Dentistry being the youngest of the learned professions—but a generation removed from its birth in science, its history has not the fascination of antiquity.

However, if a glimpse through the labyrinth of its past is not especially alluring, it should be sought for, and valued as profitable knowledge, as by comparison with vicissitudes attending its growth, we can better appreciate the present and find encouragement for the future; such information engenders interest and loyalty, tending to strengthen love and fidelity, to maintain inviolate the sacred trust bequeathed to us.

Allusions to the beauty and deformity of the teeth as bearing upon the physical perfection of our race can be traced through all the writings of the ancient poets and satirists, including the author of the Song of Songs.

Every century has produced dessertations and treatises relating to the teeth. History has not authenticated that art corrected their defects to any extent, in those ages. The oft repeated statement that the jaws of nummies gave evidence of expert dental skill among the ancient Egyptians, is entirely fabulous. Eustachius gave their anatomy in 1574.

The diary of the Rev. John Ward, vicar of Stratford-on-Avon from 1648 to 1679, containing also reminiscences of Shakespeare, reads as follows: "Upon a signe about Fleet Bridge this is written."

Here lives Peter de la Roch and George Goslin, both which, and no other, are sworn Operators to the King's teeth."

This being the earliest evidence of special dental operators.

A retrospective span of fifty-six years marks the event of the birth of dentistry, as now recognized, America being its cradle.

Since that epoch its wheels of progress have turned with such increasing rapidity from decade to decade, that to try all things and to hold fast to the good—to keep pace with the vanguard—has enforced our utmost activity.

Innovation following innovation has familiarized us with the marvelous. The crowning event of the century being the World's Columbian Dental Congress—the greatest, most auspicious, instructive and in effect far-reaching in our history. Who did not feel a thrill of pride on that occasion in being one of a profession whose achievement had been so great, and then for the first time presented for the world's recognition?

To the dental profession of Chicago our homage is due for so worthily sustaining their part as host of the dental world. In a research of our history of fifty-six years for a single event one most likely to challenge our attention and interest, with an added practical sequel, one has been chosen which was then hailed as "a consummation devoutly to be wished." as a happy alternative: as previously the forceps or turn-key had been alone indicated for treatment in pains of odontalgia.

Its consideration by this assembly is peculiarly fitting as the semi-centennial of its advent—

Without historical knowledge, the recent graduate is liable to regard the invention of crown and bridge-work as the grand division line between ancient and modern dentistry: while those of more extended practice will cite the discovery of coffer dam and the dental engine as the period of separation between the false and the true; those of still more remote matriculation quote pulp-capping, root-filling and cohesive gold-foil as the first helpful light to their professional pathway.

He only of maturer years can lead you in reminiscence to the welcome announcement of the discovery of atmospheric pressure for retention of dental plates and Hullihen's operation for the silencing of aching teeth.

Realizing how circumscribed were the resources of that time, we can comprehend the enthusiasm attending this revelation—that dental plates would hang upon their own hook, and throbbing pulps disposed of with a simple twist of the drill.

No subsequent discovery in our existence has been proclaimed with more joy than that with which the glad tidings of this event rang out just fifty years ago. At that time Dr. S. P. Hullihen, an eminent dentist, of Wheeling, Virginia, discovered an operation for the surgical treatment of dental pulps which he denominated "Rhisodontrypy," a description of the operation being to drill through the gum and alveoli a line within the margin of the latter, into the pulp canal.

This unique operation was for exposed or congested pulps, either previous or subsequent to the operation of filling. In cases where life remained, the purpose was to give vent only, wounding the pulp as slightly as possible—aiming to preserve its vitality—the gum acting as a valve for its further protection. The drill was usually driven with a bow and slack cord.

The American Society of dental Surgeons, and dental journals of that time discussed its practicability with little adverse criticism.

During its period of popularity (a seven years' wonder), as with inventors at the present time, many claimants clamored for the honor of priority, in a discovery so promising, among which were Drs. Hale, of St. Louis; John S. Clark, of New Orleans, and S. P. Miller, of Worchester, Mass; all admitting that Dr. Hullihen first made the discovery public.

At the thirteenth annual meeting of the American Society of Dental Surgeons, held at Newport, Rhode Island, August 3d 1852, Dr. C. O. Cone, of Baltimore, first brought the attention of the profession generally to the discovery of Dr. Hullihen, accompanied by a paper on the subject from the latter, stating the length of time it had been tested. The subject was discussed, and regarded as one of the most important steps to which the profession had advanced.

In the same year (1852), the *Dental Register of the West*, editorially said: "Dr. Hullihen, of Wheeling, Va., has introduced a new method of treating these delicate and sensitive threads of vitality which bids fair to supersede the killing method—they are bled into subjection and appear to behave much better than when poisened to death. The mere fact of preserving the vitality of the teeth and also the national color of the organ is sufficient to warrant a general introduction of the plan."

Dr. John S. Clark, of New Orleans, writes to the *Dental Register* in April, 1853, as follows: "I have usually a half dozen cases under treatment, and more at the present writing. As to the honors in the new operation, I say God-speed to the man who can advance the profession one step toward 'that good time coming.'"

The foregoing fragment of dental history, perchance, would not have been reviewed for your edification in historical lore alone—the dust would not have been wiped from generally discarded and forgotten methods, had we not an example of history repeating itself—but for the aid its sequel brings in the solution of the problem. How can we best preserve deciduous teeth?

The early professional life of the writer was contemporary with the later one of Dr. Hullihen, and the operation which bore his name was employed until pulp extirpation and root-filling supplanted it.

In the treatment of deciduous teeth with pulp complications, the operation of Rhisodontrypy, with modifications, was several years since readopted with more satisfactory results than with other treatment.

The modus operandi consists in thoroughly removing pulp tissue or its debris, when exposed, when the cervix is perforated on the mesio-buccal surface one sixteenth of an inch within the gingival border and terminating at the floor of the pulp chamber; the cavity being prepared, a disc of lead properly adapted is placed on the cavity floor to prevent obstruction to the vent which the perforation affords. When filling completes the work thus treated the organ of resorption performs its natural function, without interruption and childhood is dentally blessed to the limit of nature's plan.

In the treatment of pulpitis in permanent teeth, were extreme inflammation prevails, the practice of risodontrypy can be employed to great advantage over the method of drilling through crowns or fillings, as with it the minimum of vibration results.

As we stand upon the brink of another century, contemplating its needs and possibilities, let us hope that future dental historians may profitably wipe the dust from our record in this the closing decade of the nineteenth century.

Plates, of which the accompanying miniature illustration is a representation,, will be shown to elucidate the subject. Also deciduous teeth which have been treated as described, and years subsequent normally shed, will be shown in proof of the efficacy of Rhisodontrypy.

DISCUSSION.

Dr. J. Taft: I am gratified that Dr. Brown has presented this subject, which, as he has intimated, has well nigh passed from the memory of the profession. A few remember something of its introduction, some half a century ago. When it was properly and judiciously performed, it resulted well, as we shall see from the testimony of those who were most engaged in it.

The earliest account of the operation is given by Dr. Hullihen himself, in 1845. He practiced it from that time until his death, a number of years afterward, and as he himself declared, with as good success as any other dental operation, perhaps the extraction of teeth excepted.

The operation consisted in simply perforating the root of a tooth near the border of the alveolus into its pulp chamber, wounding the pulp, thereby depleting it and causing its contraction. This was performed in teeth having pulps exposed by decay. Those having been the cause of pain were treated in this way, in a great many instances with decided success, as he affirms. He preferred, however, taking those cases that had become as little as possible the subject of disease, inflammation or aching, the result being more promising. Where the pulp was diseased, suppurating, losing its vitality, the case was of course impracticable. But in most instances where the pulp was exposed by excavation, or where it was almost exposed, but covered with a very thin layer of undecayed dentine, this operation was employed, and this was the most favorable class of cases for its employment.

This operation was brought to the attention of the public more especially and pronouncedly at the meeting of the American Society of Dental Surgeons, held in Newport, R. I., in August, 1852. At that meeting Dr. Hullihen presented the method of performing the operation. It was regarded as a very decided step of progress in the treatment of exposed pulps. Prior to that time the only resort for exposed and aching pulps was the forceps. There had been before that an effort on the part of some to preserve exposed pulps by capping, but the facilities for that operation were then so circumscribed that very little was accomplished, compared with what is now attainable by that method. Dr. Hullihen, recognizing the importance of saving alive the pulps of the teeth, performed this operation. It was attempted by a great many other members of the profession, with varying success. Dr. Hullihen, in his presentation, emphasized the importance of careful discrimination in the selection of cases, and regulated his methods of procedure according to those indications, speaking of the different individuals, pointing out the fact that the same individual at different times and under different conditions of the organism, would be differently susceptible to such operations as this.

Quite a number in the profession took up the operation with varying results, there being about the same variation in results as we find now-a-days in the performance of difficult operations.

At the time of this meeting at Newport, Dr. Hullihen claimed that he had performed the operation five hundred times with very few failures. Dr. C. O. Cone, then of Baltimore, Md., and subsequently of Lexington, Ky., took up the operation, went to Dr. Hullihen, learned his method carefully, and made a report at Newport in regard to the matter. In that report he gave an account of fifty cases which he claimed had nearly all been successful. Of course there are different ideas of success. If you make fillings that remain and prevent recurrence of decay for 10, 15, 20 or 30 years, such operations are regarded as successful, and the greater the length of time the greater the measure of success.

And so with other operations. But the physician treats a case and restores the patient to health. The next week the patient is sick again. We do not dare say, therefore, that the treatment was not a success, because the disease returned again."

Prior to this meeting at Newport, Dr. Cone addressed a letter to Dr. Hullihen, asking him certain questions in regard to this operation, as follows:

1. "Give a history of the origin of your operation for the treatment of exposed dental nerves.

2. A detailed description of the operation, the cautions to be observed in the performance of the same, and the instruments employed.

3. The symptoms attending and following the operation.

4. Indications and counter-indications for the operation.

5. Relative success and failure of the operation in general, and in different classes of teeth, and in the same mouth, at different ages.

6. Pathological changes dependent on, and effected by, the operation."

Of course Dr. Hullihen's reply, setting forth his own claims for this operation, ought to come to us with considerable force. Dr. Hullihen says:

1. "The history of the origin of the operation to which you refer is briefly this: In 1845 I was called upon to plug a molar tooth for a lady, in which the nerve was very much exposed, and under circumstances that made it impracticable at that time to attempt the destruction of the nerve in the usual way. I therefore drilled a hole into the nerve cavity of the tooth, with the view of permitting the matter to escape, should the nerve suppurate (a process I felt sure would take place very speedily), and then plugged the tooth without any reference to the pressure that the plug might make upon the nerve. It was observed both by the lady and myself that the insertion of the plug did not occasion the slightest pain. In 1846 the lady again called to have her mouth prepared for a whole upper set of artificial teeth. She informed me that the tooth that I had plugged for her, 15 months before, had never caused her the slightest pain or uneasiness.

Upon extracting the tooth, I found the fangs in a perfectly healthy condition. On breaking the tooth, I found the nerve somewhat diminished in size, but in all other respects in a healthy state. The hole which I had drilled into the nerve cavity was filling up with some osseous deposit at both ends; more at the end next the nerve than that next the gum. There was likewise some appearance of an osseous deposit at the bottom of the carious cavity.

The case immediately opened the way to a number of experiments, tending, if possible, to discover the best course of treatment in all cases where the nerve had become exposed, and that without destroying the nerve or protecting it from the pressure of the plug, causing but little, if any, pain to the patient during the operation, and without endangering any painful condition of the tooth to arise afterwards, or any discoloration to take place in it, more than is common in teeth that are plugged, and that too where the nerve is in no way exposed.

2. The operation consists in making a hole through the gum, the outer edge of the alveolar process and root of the tooth in the nerve cavity, and then in opening the blood vessel of the nerve. The hole should be made of about the caliber of the nerve, at the point operated upon. If the drill employed be too large, there will be a difficulity in determining the exact moment when the nerve is reached. If too small, in obtaining the necessary discharge of blood. The drill should be spear-shaped, one cutting edge longer than the other, spring-tempered, and having a small neck. Spear-shaped, because the point is more easily located at the place desired. One cutting edge longer than the other, because such a shaped drill gives indication of its approach to the inner cavity by catching in it before it breaks through into the cavity. Spring-tempered, because less likely to break. Small-necked, so as to permit the free escape of the cuttings made in the process of drilling. The operation may be commenced on either the incisors, cuspidata or bicuspids, by pushing the drill through the gum, down to the alveolar process, about a line back from the edge of the process and directly over the center of the root of the tooth to be operated upon. Upon the molars so that the hole will be freely opened upon the main body of the nerve. The drill is then driven forward by means of a very slack string and weak bow, until its near approach to the cavity is recognized by the catching sensation before mentioned. The drill and bow laid aside, and all the cuttings of the drill most certainly removed from the hole, then with a drill rotated by the fingers the hole may be opened into the cavity. The friction of the drill upon the gum will prevent the bleeding from it. The entrance of the drill into the nerve cavity generally opens the blood-vessels, which may at once be recognized by the color (arterial blood), and by the freedom of the discharge. By pressing a lock of cotton down into the carious cavity, an oscillation may be seen in the hole through the gum. By pressing the tooth into the alveolar cell, the bleeding may be much increased, either of which indicates (so far as the making of the opening into the nerve cavity is concerned) may be considered complete.

3. The symptoms attending the operation are, of course, the prick of the drill upon passing it through the gum; then a momentary tenderness when the drill emerges from the alveolar process into the root, then a slight, painful sensation as the drill nears the nerve, which is gradually increased until the drill is plunged into the nerve cavity; and strange as it may appear, the pain occasioned by passing the drill into the nerve cavity is never half so painful as the mere touching of the nerve through a carious cavity in a tooth. The symptoms after the operation are: first, a slight, dull pain from half to one minute in duration, after the blood begins to escape from the nerve cavity. The insertion of a plug upon a nerve scarcely ever occasions the slighest uneasiness at the time of filling the carious cavity, nor afterward, unless the opening made through the gum into the root becomes permanently closed by the cuttings of the drill or a clot of blood, and in this event the pain is instantly relieved by freeing the opening. There is always more or less soreness of the gum after the operation, but never any soreness of the tooth. This soreness of the gum never causes it to become swollen and it appears to be occasioned solely by the presence of the drill cuttings left in the hole, or from cuttings being pressed into the substance of the gum itself, from using a drill having too large a stem or neck. This kind of foreign matter often gives rise to a small pustule which forms around the hole made through the gum, which, of course, will continue to exist until the cuttings are thrown off by suppuration or otherwise removed. Sometimes, but very rarely, a small red pimple shows itself in the opening made through the gum, which pimple, from its great vascularity, appears to arise from the ruptured blood-vessels of the nerve. The slightest pressure upon it occasions a very pungent pain in the tooth. This little growth is readily destroyed by applying it to nitras argenti. One application is generally sufficient to effect a cure. But in the great majority of cases, where the operation has been properly performed, there is no soreness of the gum, nor even any appearance of the opening made through it after the first week or ten days from the time the operation has been performed.

4. The indications for performing the operation are, in all cases, where the nerve has become fairly exposed, particularly so in the teeth of young subjects, and where the pressure of a plug will likely provoke inflammation in the nerve by its close proximity to it. The counter-indications are, when the nerve is more or less inflamed, in other words, when the tooth is aching, and when from the age of the patient and appearance of the tooth there is no reason to believe that the smallness of the nerve is such that no fear of inflammation may be entertained from the insertion of a plug in the carious cavity

5. The success of the operation, when properly performed, so far as I have been able to form an opinion, may be said to be universal. Out of not less of five hundred times that I have performed the operation, during the last six years, particularly so when performed in the manner I have just described, I have yet to meet the first case where the tooth has ached, an abscess formed, or where a tooth has become necrosed in consequence of the operation. But when the operation has been improperly done, such as performing it on an aching tooth, or by making too small a hole to permit the necessary discharge of blood, or in suffering a proper-sized hole to remain choked with drill-cuttings or a clot of blood, or by breaking a drill in the nerve cavity, or in carelessly pushing a portion of gold from the carious cavity into that of the nerve—in all such cases inflammation of the nerve was sure to ensue, causing toothache, oftentimes alveolar abscess, as well as total necrosis of the tooth.

6. Your question respecting the pathological changes that may be produced in the nerve of the tooth by performance of the operation, I do not feel prepared at this time to answer. The most careful examination of many cases, and at different periods after the operation has been performed, is the only reliable way of obtaining correct information upon this subject. This kind of an investigation I have not had an opportunity to make, except to a too limited extent, to venture an opinion."

Dr. Hullihen certainly operated under very unfavorable conditions, compared with those under which we operate to-day. If we should attempt to use the bow and drill, we should find our operations very much circumscribed. The statement as to the results attained seems marvelous.

The point brought out by Dr. Brown is a very valuable one, namely, the drilling into the roots of temporary teeth, and usually with decidedly good effect. We drill into the roots of other teeth as well. I have occasionally performed this Hullihen operation, not frequently, not perhaps as frequently as I ought. It is an operation which might be employed many times by the intelligent, skillful operator, with decidedly good results. To those who care nothing about the operation, to those who are not readily able to make close discrimination and diagnosis, I would say, let it alone. Of course we now have facilities for performing this operation that Dr. Hullihen never dreamed of. We have the dental engine, which can be delicately and skillfully used for such purposes. When the pulp chamber has been nearly reached—indicated by the first pain felt by the patient—the drill can be taken in the hand, then move on until the pulp canal is opened.

This operation, like many others, has passed from the minds of the profession and perhaps has been regarded by some who are not able to perform it rightly, as a failure, but it can in many cases be employed with good results, especially when conditions are favorable.

I wish simply to explain that the presentation I have here made is based upon the fact that Dr. Brown gave his paper the title of "Rhisodontrypy." In one sense that is a correct nomination, but from the impression I received sometime ago, I thought perhaps he was going more at length into the subject of Rhisodontrypy than he did. I only brought this matter before the society on account of its historical interest. You cannot brush away the testimony of such men as I have quoted, It does not do to say that Dr. Hullihen, Dr. Cartright, Dr. Bellisario, Dr. Hume, Dr. C. O. Cone, and others, than whom none stood higher in their day, were ninnies and fools. It must not be understood that I champion the general employment of this operation; but after all, there is an education in studying old methods, sometimes; and to say, you stink, and your operations smell badly, and all that, does not amount to anything. That is not scientific. Lazarus did not stink after all. That statement was about as accurate as some others that have been made.

Dr. Barrett: Is this a miraculous operation?

Dr Taft: No, sir. The operation that Dr. Brown has presented is a feasible one, the statement of Dr. McKellops to the contrary notwithstanding; there is something in it; and to seal up the cavity, as he does, that is the true way to avoid inflammation; and by making this drainage, this escape provides against future trouble in the roots of the temporary teeth. I know it is practicable, for I have used the same method, time and again, with good results.

